# Genetic Variants of the *BAFF* Gene and Risk of Fatigue Among Patients With Primary Sjögren’s Syndrome

**DOI:** 10.3389/fimmu.2022.836824

**Published:** 2022-03-15

**Authors:** Christina-Maria Flessa, Evangelia Zampeli, Maria-Eleftheria Evangelopoulos, Vasilis Natsis, Iris L. A. Bodewes, Erika Huijser, Marjan A. Versnel, Haralampos M. Moutsopoulos, Clio P. Mavragani

**Affiliations:** ^1^ Department of Physiology, Medical School, National and Kapodistrian University of Athens, Athens, Greece; ^2^ Department of Rheumatology, Iaso General Hospital, Athens, Greece; ^3^ 1^st^ Department of Neurology, Multiple Sclerosis and Demyelinating Disease Unit, Eginition University Hospital, National and Kapodistrian University of Athens, Athens, Greece; ^4^ Department of Immunology, Erasmus Medical Center, University Medical Center Rotterdam, Rotterdam, Netherlands; ^5^ Chair Medical Sciences, Immunology, Academy of Athens, Athens, Greece; ^6^ Fourth Department of Internal Medicine, Rheumatology and Clinical Immunology Unit, “Attikon” University Hospital, Medical School, National and Kapodistrian University of Athens, Athens, Greece; ^7^ Joint Academic Rheumatology Program, National and Kapodistrian University of Athens, School of Medicine, Athens, Greece

**Keywords:** Sjögren’s Syndrome, fatigue, *BAFF*, single nucleotide polymorphisms (SNPs), rs9514828

## Abstract

**Background/Purpose:**

Primary Sjögren’s Syndrome (SS) is characterized by B lymphocyte hyperactivity with B cell activating factor (BAFF) acting as an important regulator. Single Nucleotide Polymorphisms (SNPs) of the *BAFF* gene have been implicated in the pathogenesis of several autoimmune diseases characterized by heightened fatigue levels, including primary SS. We aimed to explore potential associations between *BAFF* SNPs and fatigue status of primary SS patients.

**Methods:**

Fatigue status was assessed in 199 consecutive primary SS patients (Greek cohort) using the Functional Assessment of Chronic Illness Therapy-Fatigue (FACIT-F) scale. Clinical, histological, laboratory, psychometric and personality data were also collected. DNA extracted from peripheral blood of all patients underwent evaluation for the presence of five *BAFF* SNPs (rs9514827, rs1041569, rs9514828, rs1224141, rs12583006) by PCR. To confirm our findings, an independent replicative cohort of 62 primary SS patients (Dutch cohort) was implemented. Finally, 52 multiple sclerosis (MS) patients were served as disease controls (MS cohort). Analysis of *BAFF* SNPs in association with fatigue levels was performed by the online platforms SNPStats and SHEsis and the SPSS 26 and Graph Pad Prism 8.00 software.

**Results:**

TT genotype of the rs9514828 *BAFF* polymorphism was significantly less frequent in the fatigued primary SS patients of the Greek cohort compared to the non-fatigued (14.1% vs 33.3%). The corresponding ORs [95%CI] in the dominant and overdominant models were 0.33 [0.15-0.72], p=0.003 and 0.42 [0.23-0.78], p=0.005 respectively. The association remained significant after adjustment for the variables contributing to fatigue in the univariate analysis (OR [95% CI]: 0.3 [0.1-0.9], p=0.026). Accordingly, in the Dutch cohort, there was a trend of lower mental fatigue among patients carrying the TT rs9514828 *BAFF* genotype compared to their CC counterparts (4.1 ± 2.4 vs 6.0 ± 2.2 respectively, p=0.06). The rs9514828 *BAFF* SNP was not significantly associated with fatigue in the MS cohort.

**Conclusions:**

We report a novel association between genetic makeup and primary SS-associated fatigue with the rs9514828 TT genotype decreasing the likelihood of fatigue development among these patients. These findings need validation in multi-center studies.

## Introduction

Primary Sjögren’s syndrome (SS) is an autoimmune disease characterized by lymphocytic infiltration of the exocrine glands (mainly lacrimal and salivary) resulting in reduced secretory function and consequently to ocular and oral dryness (1). The disease affects mainly middle-aged women (1) and beyond dryness, is often manifested by extraglandular symptoms (2). Fatigue is one of those patient complaints which differs from ordinary tiredness and is described as “an ever‐present, fluctuating, and non-relievable lack of energy, being beyond one’s own control” (3). It is among the most bothersome primary SS symptoms that undermines the quality of life leading to reduced capacity to carry out a wide range of everyday activities compared to age- and sex- matched healthy control individuals (4). While alleviation of fatigability is of major importance for the majority of primary SS patients, it is often difficult to manage (5). The prevalence of fatigue among primary SS patients ranges between 38-88% (6, 7), with most studies reporting a prevalence of around 70% (8–10). These rates are striking, especially if they are compared to the prevalence of fatigue in the general population in the form of chronic fatigue syndrome, which ranges between 0.007 and 2.8% (11). Fatigue predominates in primary SS patients with arthralgias/myalgias and fibromyalgia, indicating an association with fibromyalgia (6, 7). Fatigue is also more prevalent in primary SS patients under the use of hydroxychloroquine (6) and in functionally impaired SS patients (4). Fatigued patients display statistically significant decreased type I IFN score compared to non-fatigued (6). Fatigue has been also observed in other organ specific autoimmune disorders including multiple sclerosis (MS). Indeed, fatigue is a very common symptom in MS, with almost 80% of the patients being affected; 55% of them describing it as one of the worst symptoms associated with their disease (12–14).

Several mechanisms have been implicated in fatigue pathogenesis in the setting of inflammatory rheumatic diseases, such as immunological mechanisms, central and autonomic nervous system dysfunctional changes, neuroendocrine alterations, sleep and metabolic disturbances, cardiopulmonary fitness as well as psychosocial factors (15). The pathophysiology of SS-related fatigue is multifactorial and not yet fully elucidated (16). It has been previously associated with several psychological and physiological factors, which include sleep disturbances (17–19), dysfunction of the autonomic nervous system (20, 21), depression (6, 8, 19, 22–25), dysfunctional or alexithymic psychological profile (26), anxiety (24), pain (19, 27), neuroticism and fibromyalgia (6, 28). SS related fatigue has been also associated with lower levels of proinflammatory cytokines (22), as well as with higher heat shock protein levels, through their signaling in the central nervous system (29). Transcriptional analysis revealed 19 sets of genes associated with actin filaments, migration of cells and guanine nucleotide binding protein (G-protein) signaling to be significant contributors to fatigue development in the context of primary SS (30). Along the same lines, a novel set of serum proteins named as “fatigue signature” was found by aptamer-based proteomics technology to be able to distinguish primary SS patients with fatigue from those without (31). Moreover, a recent proteomic study on the cerebrospinal fluid of primary SS patients with low and high fatigue levels identified several discriminatory proteins as determinants of severe depression and/or appetite loss (32).

Previous data have shown that targeting BAFF through the monoclonal antibody belimumab, improves fatigue levels in patients with primary SS (33, 34) and systemic lupus erythematosus (SLE) (35–37). Moreover, there are studies that recognize the neuropsychiatric adverse effects of suicidal ideation and depression in the setting of belimumab administration (38, 39). Of interest, primary SS patients have been previously described as having statistically significant higher levels of introverted hostility and feelings of guilt, somatization, anxiety, depression and obsessive compulsiveness compared to healthy controls (40). In addition, higher scores of paranoid ideation approaching statistical significance were observed between primary SS patients compared to healthy controls (40). Based on the above findings along with the previously described associations between the *BAFF/BAFF-R* axis genetic variants and primary SS (41, 42), we aimed to investigate whether variations of the *BAFF* gene could predispose to fatigue development among patients with primary SS. Furthermore, since *BAFF* variants have been previously associated with MS- also characterized by high fatigue levels- (43), MS patients were used as disease controls.

## Patients and Methods

### Patients

199 consecutive primary SS patients were evaluated at the Molecular Physiology and Clinical Applications Unit, Department of Physiology, National and Kapodistrian University of Athens (Greek cohort). Patients were followed and referred by HMM, and CPM. Furthermore, an independent primary SS patient cohort of 62 patients, followed in the Department of Immunology, Erasmus MC, University Medical Centre Rotterdam, Netherlands, was also included (Dutch cohort). All patients, Greek and Dutch, fulfilled the 2016 American/European classification criteria for primary SS (44), and were fully evaluated for fatigue, using specific questionnaires. 52 MS patients fulfilling the 2017 MacDonald classification criteria (45), followed in the Demyelinating diseases Unit, Department of Neurology, Eginition Hospital, Athens, Greece (M.E.E), also evaluated for fatigue, served as disease controls (MS cohort). All patients provided informed consent prior to the entry in the study. The study protocol was approved by the Medical Ethics Committees of the National and Kapodistrian University of Athens and of the Erasmus MC.

### Clinical, Serological and Histopathologic Data

Clinical, serological and histopathologic data of the primary SS patients (Greek cohort) were recorded after thorough medical records review. These included the presence of arthralgias-myalgias, arthritis, subjective measures of oral and ocular dryness, salivary gland enlargement, Raynaud’s phenomenon, palpable purpura and lymphoma development. Additionally, laboratory data including white blood cell (WBC) counts/erythrocyte sedimentation rate (ESR), hemoglobin (HGB), thyroid stimulating hormone (TSH) and lactate dehydrogenase (LDH) levels were recorded. Antinuclear antibodies (ANAs), anti-Ro/SSA, anti-La/SSB, rheumatoid factor (RF), serum complement and immunoglobulin levels, the presence of hypergammaglobulinemia, abnormal Schirmer’s test and ocular stain positivity as well as histopathologic characteristics including the noted Tarpley and focus (number of foci/4mm^2^) scores were also recorded. The European League Against Rheumatism (EULAR) Sjögren’s Syndrome Disease Activity Index (ESSDAI) score was also calculated (46).

Data about the age, sex and Expanded Disability Status Scale (EDSS) (a method of quantifying disability in MS and monitoring changes in the level of disability over time) of the MS patients were also documented (47).

### Psychometric Scales

#### Greek Primary SS Cohort

Psychological features of primary SS patients (Greek cohort) were assessed using the following self-administered psychometric questionnaires: 1) State-Trait Anxiety Inventory (STAI) (48), a questionnaire used to assess anxiety either as a personality feature or as a current condition; 2) Eysenck Personality Questionnaire (EPQ) Scale (49), a questionnaire that estimates personality traits and temperamental aspects of behavior based on the three independent axes of neuroticism, psychoticism, and extroversion; 3) Zung Depression Scale (50), a validated tool for assessing depression; and 4) Athens Insomnia Scale (AIS) (51), a questionnaire that assesses sleep disturbances. The cutoff value is >35 for the STAI scale, >12 for the EPQ neuroticism, >2 for the EPQ psychoticism, >9 for the EPQ extroversion, >40 for the Zung Depression Scale and >6 for the AIS. The aforementioned questionnaires have been validated for the Greek population and have been previously used for psychiatric assessment in autoimmune patients. Cutoff points were defined following the normative data derived from the validation of scales within the Greek population (52).

In order to assess fatigue in our study population, the Functional Assessment of Chronic Illness Therapy-Fatigue (FACIT-F) scale was used. This scale was originally developed in order to assess fatigue in cancer patients with or without anemia, compared to the general US population (53) and it has been validated in the general population (54) and has been also largely used and validated in patients with inflammatory (55–57) and autoimmune diseases, including primary SS (58–61). The score of the FACIT-F scale ranges from 0 to 52, higher values reflect lower fatigue levels and a cutoff score of <30 indicates severe fatigue (62). According to this cutoff point, primary SS patients were classified as fatigued (FACIT-F score <30) and non-fatigued (≥30 in the FACIT-F scale).

#### Dutch Primary SS Cohort

The Dutch cohort adopted the Multidimensional Fatigue Index-20 (MFI-20) for assessing fatigue in five dimensions, namely general fatigue, physical fatigue, reduced activity, reduced motivation and mental fatigue (63) and the EULAR SS patient-reported index (ESSPRI) for assessing global and mental fatigue together with pain and dryness experienced by patients (5). There were 4 different models of the ESSPRI scale that were tested for their correlation with the patient reported evaluation of the global severity of their symptoms related to their primary SS (PGA) and the one mostly correlated (including dryness, limb pain and fatigue) was finally implemented as ESSPRI (5). However, since mental fatigue has been found to be worse in primary SS patients compared to healthy controls (64), model 2 of the ESSPRI which includes dryness, fatigue, pain and mental fatigue has been chosen for the evaluation of the patients included in the Dutch cohort. In the MFI-20 scale there is no cut-off point to indicate the severity of fatigue and the authors argue against using a total score derived from adding the five dimensions, so in this scale higher scores indicate a higher fatigue level (63). In the ESSPRI scale there is no single cut-off point either and each domain represents the symptom severity independently, although it is also possible to obtain a total score from the average of the four domains (5). Higher score represents more severe symptoms and a patient-acceptable symptom state (PASS) estimate has been created which is defined as an ESSPRI <5 points (65).

#### MS Cohort

Fatigue in the MS cohort was assessed with the Fatigue Severity Scale (FSS) and the Fatigue Scale for Motor and Cognitive Functions (FSMC) (66, 67). The FSS is a nine-item scale that measures fatigue severity and how much it affects the patient’s activities and lifestyle. Severe fatigue was defined as an FSS score of 5 or higher (68). The FSMC was developed for the assessment of MS-related cognitive and motor fatigue (67). It consists of 20 items, 10 referring to cognitive and 10 to physical fatigue. A higher score in each subscale and the total scale indicates higher levels of fatigue, while according to cut-off values patients can be categorized as having mild, moderate or severe fatigue (67).

### *BAFF* Genotyping, *BAFF* Gene Expression and BAFF Serum Levels

Blood samples for DNA and RNA extraction were collected from all subjects of the Greek primary SS and of the MS cohorts using ethylenediamine tetraacetic acid (EDTA) tubes. Blood samples from the patients of the Dutch cohort were collected in sodium-heparin tubes and the nucleated cells were isolated by centrifugation. Serum samples were also collected in serum tubes with clot activator and stored at -80°C. Genomic DNA from the patients of the Greek primary SS and of the MS cohorts was extracted from the collected blood samples using the Nucleospin Blood QuickPure kit (Macherey-Nagel GmbH & Co, Germany), according to the manufacturer’s instructions. Genomic DNA from the patients of the Dutch cohort was extracted from the isolated nucleated cells using the DNeasy Blood & Tissue Kit (QIAGEN, Germany), according to the manufacturer’s protocol. Total RNA was isolated with the TRIzol™ Reagent according to the manufacturer’s instructions (Invitrogen, USA). DNA and RNA concentrations were measured by Biospec-Nano spectrophotometer (Shimadzu, Japan).

We examined five *BAFF* SNPs previously reported in primary SS patients (41). Three SNPs were located in the 5’ regulatory region within 5kb of the first exon of the *BAFF* gene (*TNFSF13B*, chromosome 13, 107 715_107 725K) and included the rs9514827 (c.-2841 T > C), the rs1041569 (c.-2701 A > T), and the rs9514828 (c.-871 C > T). Additionally, two SNPs located in the noncoding region of *TNFSF13B* flanking exon 3 were also tested and included the rs1224141 (T > G) and the rs12583006 (T > A). These five SNPs of the *BAFF* gene were evaluated by PCR-based assays as previously described (41).

We also performed complementary DNA (cDNA) synthesis from the total RNA extracted and measured *BAFF* gene expression levels in 44 primary SS patients from the Greek cohort by real-time quantitative polymerase chain reaction (qPCR) as previously described (6). *Glyceraldehyde-3-phosphate dehydrogenase* (*GAPDH*) was used as the reference gene used to perform relative quantification. Oligonucleotide primers used in the qPCR were as follows: BAFF, F: 5’-AGTTCAAGTAGTGATATGGATG-3’ and R: 5’-GGGAGGATGGAAACACAC-3’; and GAPDH, F: 5’-CAACGGATTTGGTCGTATT-3’ and R: 5’-GATGGCAACAATATCCACTT-3’.

Serum BAFF levels were measured using a polyclonal human BAFF enzyme linked immunosorbent assay (ELISA) kit according to the manufacturer’s protocol (Human BAFF/BLyS/TNFSF13B Quantikine ELISA Kit, R&D systems) in 87 primary SS patients of the Greek cohort. Quality controls (low, medium and high) were used to ensure the reliability and accuracy of the BAFF measurements in each plate. Appropriate dilutions of all samples were prepared in order for the samples to run within the standard curve. The Greek primary SS cohort was subdivided in two groups (high and low serum BAFF) according to a cut-off level corresponding to the upper quartile of serum BAFF levels in the cohort (1186 pg/ml). These two groups were used for an haplotype analysis and anti-CD20, hydroxychloroquine (HCQ) and steroid use treatment analysis to be performed in a search for possible associations with serum BAFF levels.

### Statistics

Allele, genotype and haplotype frequencies in primary SS patients with or without fatigue were determined for each SNP by SHEsis and SNPStats software using the chi-square (χ^2^) test (69–71). Adjusted odds ratios (OR) and corresponding 95% confidence intervals (CI) were estimated by unconditional logistic regression adjusting for the effects of age and gender. The five genetic models (codominant, dominant, recessive, overdominant and additive) were also determined (69).

Two-sided Fisher’s exact, chi-square (χ^2^), and Mann-Whitney tests were implemented for the comparison of qualitative and quantitative characteristics, respectively, between primary SS patients with and without fatigue (SPSS 26 statistical software, IBM and Graph Pad Prism 8.00, Graph Pad Software). To assess whether *BAFF* variants are independently associated with fatigue, a multivariate model was constructed by backward stepwise conditional logistic regression, considering all variables that turned out to be significant in univariate analysis. Furthermore, logistic regression was also applied to assess whether *BAFF* variants are associated with fatigue independently of lymphoma development.

## Results

### Demographic, Clinical and Laboratory Data of Primary SS Patients and MS Disease Controls

The main demographic, clinical and laboratory characteristics of the 261 primary SS patients (199 patients in the Greek and 62 in the Dutch cohort), as well as the main patient and disease characteristics (age and gender of patients and the EDSS score)of the 52 MS disease controls included in the study are displayed in **Supplementary Table 1**.

### Demographic, Clinical and Laboratory Data of Primary SS Patients According to the Fatigue Status (Greek Cohort)

Fatigue was present in 64 of 199 (32.2%) primary SS patients. In **Supplementary Table 2**, demographic characteristics, as well as the main clinical and laboratory indices are compared in the two distinct primary SS groups according to the presence or absence of fatigue. No significant associations with any clinical or laboratory findings including ESSDAI, HGB concentration, ESR, TSH levels or lymphoma status were detected. Fatigue significantly associated with arthralgias/myalgias (76.2% of patients with fatigue experienced arthralgias/myalgias compared to 58.6% of patients without fatigue, p=0.017), marginally associated with Raynaud’s phenomenon (34.9% of the patients with fatigue are affected by Raynaud’s phenomenon compared to 22.0% of patients without fatigue, p=0.05) as well as with medication treatment. Thus, fatigued patients were more likely to receive steroids (45.3% compared to 26.7% of non-fatigued patients, p=0.009), HCQ (57.8% of patients with fatigue are under HCQ treatment compared to 41.5% of patients without fatigue, p=0.03) and anti-CD20 (28.1% of fatigued patients receive anti-CD20 medication compared to 14.1% of non-fatigued patients, p=0.02). Serum BAFF levels were associated with anti-CD20 treatment, but not with fatigue scores, HCQ or steroid use (data not shown).

### Association of Fatigue With Psychometric Parameters Within the Greek Primary SS Cohort

As shown in **Supplementary Table 3**, fatigue was found highly significantly associated with anxiety (both as state and trait), depression, impaired sleep, psychoticism, neuroticism and extroversion. Patients with fatigue present higher rates of anxiety, both as state (95.3% vs 60.9% in primary SS patients without fatigue; p<0.001) and trait (96.8% vs 70.4% in primary SS patients without fatigue; p<0.001), as well as depression (84.4% vs 51.9% in non-fatigued primary SS patients, p <0.001). Furthermore, 90.3% of primary SS patients with fatigue have impaired sleep pattern and quality as measured by the AIS scale, compared to only 41.4% of primary SS patients without fatigue (p<0.001). Psychoticism and neuroticism were also more prevalent in fatigued vs non-fatigued primary SS patients [81.3% vs 64.4%, p=0.02 and 92.2% vs 49.6%, p<0.001, respectively). Of interest, extroversion rates showed an opposite pattern with patients without fatigue being more extrovert than those with fatigue (80% vs 65.6% respectively, p=0.03). Taken together, these results confirm previous observations of studies supporting that psychological, personality and sleep-related characteristics are associated with fatigue in the setting of primary SS (6, 8, 17–28).

### Allele, Genotype and Haplotype Analysis of the *BAFF* Gene in the Greek Primary SS Cohort

**Figure 1:**
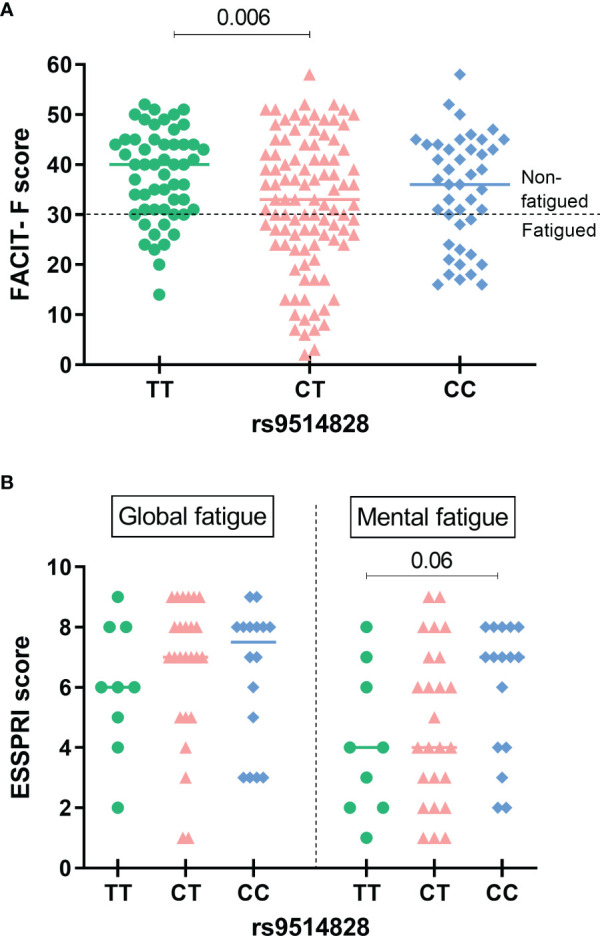
Fatigue scores according to the rs9514828 *BAFF* genotypes in the Greek and Dutch primary SS cohorts. **(A)** FACIT-F scores were sighificantly higher in patients bearing the TT genotype compared to their CT (mean ± SD: 37.7 ± 9.0 vs 32.1 ± 13.3 respectively, p=0.006), but not to their CC counterparts. The line at point 30 of the scale indicates the cut-off point for fatigue. The horizontal bars at each data set represent medians. **(B)** While no significant differences in global or mental fatigue scores of the ESSPRI were detected across different genotypes, there was a trend of lower mental fatigue scores among primary SS patients carrying the TT rs9514828 genotype compared to their CC counterparts (mean ± SD: 4.1 ± 2.4 vs 6.0 ± 2.2 respectively, p=0.06). The horizontal bars at each data set represent medians. ESSPRI, EULAR Sjogren’s Syndrome Patient Reported Index, SNP, Single Nucleotide Polymorphism, FACIT-F, Functional Assessment of Chronic Illness Therapy-Fatigue, SS, Sjögren**’**s Syndrome.

While the allele analysis for the prevalence of the five studied *BAFF* SNPs between the primary SS groups with or without fatigue revealed no statistically significant differences (**Supplementary Table 4**), the TT genotype of the rs9514828 *BAFF* SNP was less frequent in fatigued compared to non-fatigued primary SS patients (14.1% vs 33.3%, OR [95%CI]: 0.33 [0.15-0.72], p=0.003 and 0.42 [0.23-0.78], p=0.005 in the dominant and overdominant models respectively) (**Table 1**). FACIT-F scores according to rs9514828 genotypes are depicted in **Figure 1A**, with Greek primary SS patients bearing the TT genotype developing higher FACIT-F scores compared to their CT counterparts (37.7 ± 9.0 vs 32.1 ± 13.3 respectively, p=0.006). The association between TT genotype and fatigue development remained significant after adjustment for all variables shown to contribute to fatigue in the univariate analysis (arthralgias-myalgias, anxiety as a state or a trait, depression, psychoticism, neuroticism, extroversion, sleep disturbances, hydroxychloroquine, steroids and anti-CD20) (OR [95% CI]: 0.3 [0.1-0.9], p=0.026), indicating that the TT genotype of the rs9514828 SNP is independently associated with lower fatigue levels in the setting of primary SS (data not shown). Moreover, the rs9514828 TT genotype was found independently associated with lower risk for fatigue, following adjustment for lymphoma development (OR [95% CI]: 0.3 [0.1-0.7], p=0.006) (data not shown). No other associations were detected in the genotypic analysis between the other *BAFF* SNP variants (rs1224141, rs12583006, rs1041569 and rs9514827) and fatigue development. Furthermore, no statistically significant differences in the demographic, clinical and laboratory data were observed between the rs9514828 *BAFF* genotypes (**Supplementary Table 5**), indicating that the patients bearing the TT genotype differed only in their fatigue status and not in their clinical/laboratory picture compared to their CC and CT counterparts.

**Table 1:** Genotypic frequencies of five *BAFF* SNPs in primary Sjögren’s syndrome (SS) groups with or without fatigue (Greek cohort).

SNP	Genotypes	Fatigued(n=64)n (%)	Non-fatigued(n=135)n (%)	OR Codominant model [95%CI]	p- value	OR Dominant model [95%CI]	p- value	OR Recessive model [95%CI]	p- value	OR Overdominant model [95%CI]	p- value	OR Additive model [95%CI]	p- value
**rs1224141**	TT	38 (59.4)	97 (71.8)	1	0.14	1.75 [0.94-3.26]	0.08	4.32 [0.38-48.58]	0.22	1.59 [0.84-2.99]	0.15	1.76 [0.99-3.14]	0.06
	GT	24 (37.5)	37 (27.4)	1.66 [0.88-3.13]									
	GG	2 (3.1)	1 (0.7)	5.11 [0.45-57.97]									
**rs12583006**	TT	35 (54.7)	71 (52.6)	1	0.30	0.92 [0.51-1.67]	0.78	2.00 [0.73-5.45]	0.18	0.71 [0.38-1.33]	0.28	1.09 [0.69-1.73]	0.70
	TA	21 (32.8)	55 (40.7)	0.77 [0.41-1.48]									
	AA	8 (12.5)	9 (6.7)	1.80 [0.64-5.08]									
**rs9514828**	CC	13 (20.3)	30 (22.2)	1	**0.006**	**0.33** **[0.15-0.72]**	**0.003**	1.12 [0.54-2.33]	0.76	**0.42** **[0.23-0.78]**	**0.005**	1.43 [0.93-2.21]	0.10
	CT	42 (65.6)	60 (44.4)	**1.61 [1.39-1.89]**									
	TT	9 (14.1)	45 (33.3)	0.46 [0.18-1.22]									
**rs1041569**	AA	38 (59.4)	75 (55.6)	1	0.75	0.86 [0.47-1.56]	0.61	0.59 [0.12-2.92]	0.50	0.93 [0.50-1.71]	0.81	0.84 [0.50-1.41]	0.50
	AT	24 (37.5)	53 (39.3)	0.89 [0.48-1.66]									
	TT	2 (3.1)	7 (5.2)	0.56 [0.11-2.85]									
**rs9514827**	TT	30 (46.9)	63 (46.7)	1	0.98	0.99 [0.55-1.80]	0.98	0.89 [0.33-2.45]	0.83	1.03 [0.57-1.88]	0.92	0.97 [0.62-1.53]	0.90
	TC	28 (43.8)	58 (43)	1.01 [0.54-1.90]									
	CC	6 (9.4)	14 (10.4)	0.90 [0.31-2.57]									

ORs and p-values for codominant, dominant, recessive, overdominant and additive models are shown. Fatigued patients are considered those with a FACIT-F score <30 and non-fatigued those scoring ≥30 in the FACIT-F scale. The bold values indicate the values/correlations that are statistically significant.

**Figure 2:**
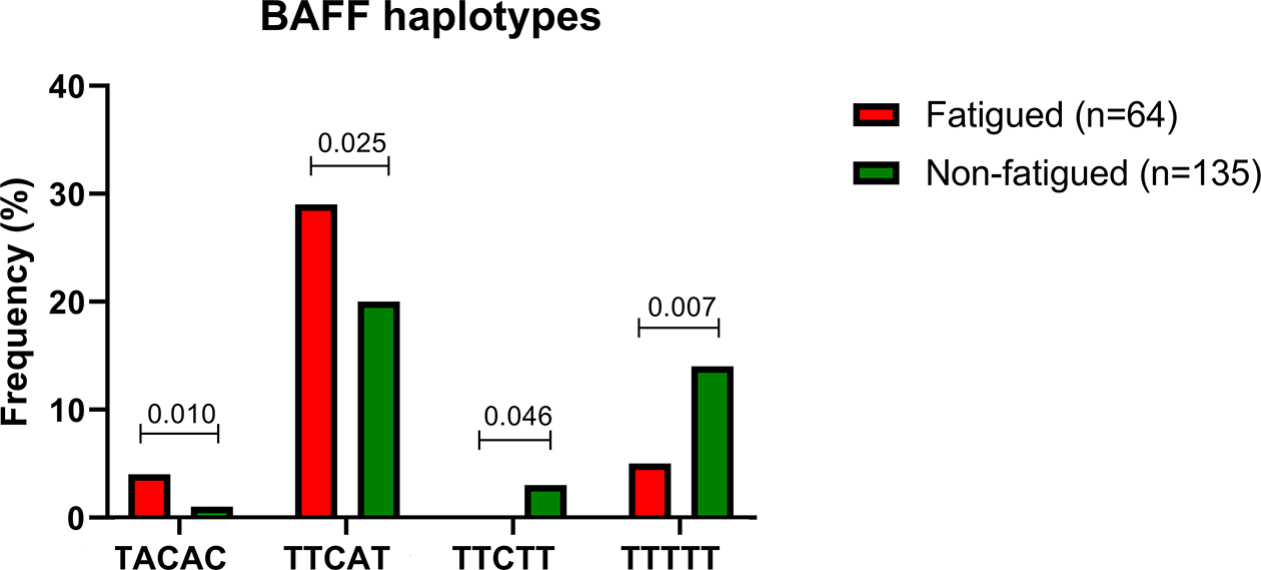
Significant differences in haplotype frequencies between fatigued and non fatigued Greek primary SS individuals are displayed.

We next sought to explore whether certain haplotypes of the *BAFF* SNP variants could be associated with fatigue development among primary SS patients. The five *BAFF* SNPs tested formed 12 common haplotypes (frequency for both groups >3%). According to haplotype analysis, the TACAC and TTCAT haplotypes (alleles for rs1224141, rs12583006, rs9514828, rs1041569 and rs9514827 respectively) were more prevalent in the fatigued primary SS group compared to non-fatigued counterparts (OR [95%CI]: 7.496 [1.264-44.458] and 1.759 [1.072-2.888], p-values: 0.010 and 0.025 respectively); in contrast TTCTT and TTTTT haplotypes were associated with decreased fatigue susceptibility among primary SS patients (OR [95%CI]: 0.000 [0.000-0.000] and 0.319 [0.134-0.761], p-values: 0.046 and 0.007, respectively) (**Table 2** and **Figure 2**). No association between TACAC, TTCAT, TTCTT or TTTTT haplotypes and serum BAFF levels was detected (data not shown).

**Table 2:** Haplotype analysis of *BAFF* SNPs rs1224141, rs12583006, rs9514828, rs1041569 and rs9514827 in primary Sjögren’s syndrome (SS) patient groups with or without fatigue (Greek cohort).

Haplotype	Fatigued (n=64) (frequency)	Non-fatigued (n=135) (frequency)	Fisher’s p value	OR [95%CI]
G T C A T*	0.03	0.05	0.425	0.624 [0.194-2.008]
G T T A C*	0.07	0.03	0.048	2.665 [0.976-7.281]
G T T T T*	0.08	0.05	0.140	1.877 [0.805-4.376]
T A C A C*	**0.04**	**0.01**	**0.010**	**7.496 [1.264-44.458]**
T A C A T*	0.10	0.11	0.865	0.942 [0.472-1.877]
T A T A C*	0.05	0.09	0.195	0.553 [0.223-1.370]
T A T T T*	0.04	0.02	0.114	2.801 [0.742-10.575]
T T C A T*	**0.29**	**0.20**	**0.025**	**1.759 [1.072-2.888]**
T T C T T*	**0.00**	**0.03**	**0.046**	**0.000 [0.000-0.000]**
T T T A C*	0.11	0.14	0.354	0.731 [0.377-1.420]
T T T A T*	0.02	0.05	0.233	0.466 [0.129-1.683]
T T T T T*	**0.05**	**0.14**	**0.007**	**0.319 [0.134-0.761]**

Fatigued patients are considered those with a FACIT-F score <30 and non-fatigued those scoring ≥30 in the FACIT-F scale. The bold values indicate the values/correlations that are statistically significant. The symbol "*" denotes all haplotypes with a frequency>0.03, since those haplotypes with frequency<0.03 were ignored in the analysis.

### Association of the *BAFF* rs9514828 Polymorphism With Fatigue Levels in the Dutch Primary SS Cohort

Given our observations supporting a “protective” role of the minor TT genotype of the *BAFF* rs9514828 polymorphism towards fatigue development, we then performed analysis of the five *BAFF* SNPs in an independent cohort of primary SS patients (Dutch cohort) and wished to explore whether there were associations between the *BAFF* SNPs and fatigue reported symptoms. In accordance with our findings of the Greek cohort, there was a trend of lower ESSPRI mental fatigue scores among primary SS patients carrying the TT rs9514828 genotype compared to their CC counterparts (4.1 ± 2.4 vs 6.0 ± 2.2 respectively, p=0.06) (**Figure 1B**). No other associations between the *BAFF* SNPs and fatigue occurrence as assessed by the MFI-20 scale total score and its five dimensions (general fatigue, physical fatigue, reduced activity, reduced motivation and mental fatigue) were detected.

### No Association of the *BAFF* rs9514828 Polymorphism With Fatigue in MS

We next wished to explore the role of the rs9514828 polymorphism in the generation of fatigue in the setting of MS, an organ specific autoimmune disease hallmarked by heightened fatigue levels (12–14). The associations of the rs9514828 *BAFF* polymorphism with the fatigue status in the MS cohort as assessed by the FSS, the FSMC cognitive, FSMC physical and FSMC total scores are summarized in **Table 3**. The genotypes of the rs9514828 SNP displayed no difference in their impact on fatigue development in the MS cohort.

**Table 3:** Associations of the rs9514828 *BAFF* polymorphism with the fatigue status of patients in the multiple sclerosis (MS) cohort as assessed by the Fatigue Severity Scale (FSS) and the Fatigue Scale for Motor and Cognitive Functions (FSMC).

	BAFF rs9514828		
	TT (n=9)	CT/CC (n=43)	p-value
**FSS score (mean ± SD)**	4.0 ± 1.3	3.9 ± 1.7	ns
**FSS score ≥5 (severe fatigue) (%)**	33.3	30.2	ns
**FSMC cognitive score (mean ± SD)**	22.8 ± 9.7	26.8 ± 10.6	ns
**FSMC physical score (mean ± SD)**	27.6 ± 8.6	29.9 ± 11.9	ns
**FSMC total score (mean ± SD)**	50.2 ± 16.2	56.3 ± 21.5	ns

ns, non-significant.

## Discussion

In the present study we aimed to investigate whether variants of the *BAFF* gene influence fatigue susceptibility among primary SS patients. To our knowledge, this is the first time that the TT genotype of the *BAFF* rs9514828 gene variant was found to be protective against fatigue development independently of other potential cofounders. While this observation was in accordance with data on the Dutch primary SS cohort and the rs9514828 TT genotype showed an inverse association with fatigue status in the two independent primary SS cohorts, no association between the rs9514828 TT genotype and fatigue occurrence was detected in the MS group.

Previous studies have revealed associations of TT genotype of the rs9514828 *BAFF* polymorphism with enhanced disease activity, serum autoantibodies, increased serum BAFF levels and lymphomagenesis in the setting of primary SS (41, 42, 72). Therefore, the lower prevalence of the TT rs9514828 genotype among fatigued primary SS patients could reflect the absence of systemic and B cell activation markers in this primary SS subset. Indeed, fatigue has been previously designated as a major component of the so called “Dryness, Pain, Fatigue (DPF)” phenotype of primary SS, which lacks severe organ dysfunction or serum autoantibodies; though it does not directly affect survival, it seriously impacts quality of life (27, 73, 74). Fatigue in primary SS denotes a low disease activity as displayed by the inverse correlation between fatigue levels and pro-inflammatory cytokines (22, 75, 76) and is also independent of B cell activation given the absence of association between fatigue and autoantibodies, serum BAFF levels and hypergammaglobulinemia (6). Interestingly, DPF features -previously shown to strongly predict quality of life- are optimally assessed by the ESSPRI rather than using ESSDAI, which reflects disease activity and systemic involvement (77, 78). Furthermore, recent data indicate that BAFF serum levels are reduced in unmedicated depressed patients compared to healthy controls and remain low during the first phase of antidepressant treatment. As the patients continue receiving antidepressant medication, BAFF levels increase (79). While the mechanistic links between low BAFF and depression remain to be elucidated, a role of BAFF in neurotransmission has been suggested (79).

To the best of our knowledge this is the first time that variants of the *BAFF* gene are associated with fatigue among primary SS patients. Some efforts have been made to identify genetic or epigenetic factors contributing to fatigue development in the context of chronic fatigue syndrome or other entities, such as cancer, hepatitis C, infectious mononucleosis, multiple sclerosis, hypothyroidism and hereditary hemochromatosis [reviewed in (80, 81)]. In the setting of primary SS, associations between fatigue and gene variations previously implicated in chronic fatigue syndrome were detected (82). More specifically, a trend for association between fatigue and one SNP in the gene *SLC25A40*, encoding a mitochondrial carrier protein in the brain and periphery, and two SNPs in the gene *PKN1*, encoding a protein kinase C superfamily member was detected (82). The latter is ubiquitously expressed and is implicated in cell signaling, apoptosis and negative regulation of the pro-inflammatory transcription factor Nuclear Factor kappa-light-chain-enhancer of activated B cells (NF-κB) (82). Furthermore, according to the results of a genome-wide association study (GWAS), genetic variants of the genetic locus *Receptor Transporter Protein 4 (RTP4)/MASP1* were found to be significantly associated with primary SS-related fatigue (83, 84). *RTP4* gene encodes a Golgi chaperone protein involved in pain processing, through the cell surface expression of opioid receptors, while *MASP1* is involved in complement activation and is specifically expressed in liver, brain, female tissues and muscular tissues (83, 84).

Additionally, another 58 SNPs in 4 genes, namely *Endoplasmatic Reticulum to Nucleus Signaling 1 (ERN1), Long intergenic non-protein coding RNA 1553 (LINC01553), Long intergenic non-protein coding RNA 1184 (LINC01184)* and *(RP11-15I11.2)* were significantly associated with primary SS-related fatigue (83). Finally, a recent epigenetic study revealed that 251 CpG sites annotated to 182 genes were differentially methylated between primary SS patients with or without fatigue; 166 sites were hypomethylated and 85 were hypermethylated in the high fatigue compared to the low fatigue group (85).

The main strengths of the current study are the large number of patients with complete clinical and psychometric evaluation, as well as the use of an independent replication primary SS group of different origin (Dutch primary SS cohort) and a disease control group (MS cohort). According to our knowledge, this is the largest so far study assessing fatigue and its genetic associations in primary SS. However, the study is not without limitations. We acknowledge that due to the study design there is heterogeneity in the scales implemented to assess fatigue levels. Moreover, the MS cohort needs to be expanded with larger patient numbers to confirm these observations.

Given the recently identified association between serum BAFF levels and autoantibodies against the NR2 subunit of N-methyl-D-aspartate receptor (NMDAR) and motor fatigue in systemic lupus erythematosus (SLE) patients (86), it would be highly interest to test whether anti-NMDAR autoantibodies could contribute to fatigue levels in primary SS patients as well.

In conclusion, we have identified a protective role of the TT genotype of the rs9514828 *BAFF* gene variant against fatigue development in primary SS patients. Interestingly, this genotype confers protection from fatigue independently of other contributing factors. This protective role was not confirmed in MS patients, probably indicating a disease-specific role of this variant. Further studies with more uniform implementation of fatigue scales in the populations tested are needed to validate our findings.

## Data Availability Statement

The data presented in the study are deposited in the European Variation Archive (EVA) repository (https://www.ebi.ac.uk/eva/), accession number PRJEB51100.

## Ethics Statement

The studies involving human participants were reviewed and approved by Ethics Committee of National and Kapodistrian University of Athens, Athens, Greece and Ethics Committee of the Erasmus MC, Rotterdam, Netherlands. The patients/participants provided their written informed consent to participate in this study.

## Author Contributions

CM conceptualized and designed the study. C-MF performed the experimental procedures. EZ, M-EE, VN, MV, IB, EH, HM, and CM provided clinical data and samples from patients with primary Sjögren’s syndrome and multiple sclerosis. CM and C-MF analyzed, interpreted data, and wrote the manuscript. M-EE, MV, and HM reviewed the manuscript. Final approval of the manuscript was given by all authors. All authors have read and agreed to the published version of the manuscript.

## Funding

This research is co-financed by Greece and the European Union (European Social Fund- ESF) through the Operational Programme «Human Resources Development, Education and Lifelong Learning» in the context of the project “Strengthening Human Resources Research Potential *via* Doctorate Research” (MIS-5000432), implemented by the State Scholarships Foundation (IKY).

## Conflict of Interest

The authors declare that the research was conducted in the absence of any commercial or financial relationships that could be construed as a potential conflict of interest.

## Publisher’s Note

All claims expressed in this article are solely those of the authors and do not necessarily represent those of their affiliated organizations, or those of the publisher, the editors and the reviewers. Any product that may be evaluated in this article, or claim that may be made by its manufacturer, is not guaranteed or endorsed by the publisher.
