# Supplementary material for: Genetic Variants of the BAFF Gene and Risk of Fatigue Among Patients With Primary Sjögren’s Syndrome
Source: Front Immunol. 2022 Mar 15;13:836824. doi: 10.3389/fimmu.2022.836824 (PMC8964489; doi:10.3389/fimmu.2022.836824)
Supplement: Supplementary file 1 [file DataSheet_1.docx]

**SUPPLEMENTARY MATERIAL**

**Supplementary Table 1.** Demographic, clinical and laboratory characteristics of the primary Sjögren's syndrome (SS) and multiple sclerosis (MS) patients included in the study.

| **Patient characteristics** | **Greek primary SS cohort (n=199)** | **Dutch primary SS cohort (n=62)** | **MS cohort (n=52)** |
| --- | --- | --- | --- |
| Age (years), median (range) | 59.5 (15-85) | 61 (20-92) | 38.5 (19-58) |
| Female sex (%) | 94.5 | 93.5 | 61.5 |
| Age at primary SS/MS diagnosis, median (range) | 52 (15-81) | 47.5 (18-73) | 36 (16-50) |
| Dry mouth subjective (%) | 92.9 | N/A | N/A |
| Dry eyes subjective (%) | 90.9 | N/A | N/A |
| Salivary gland enlargement (%) | 29.2 | N/A | N/A |
| Abnormal Schirmer's test (%) | 76.7 | N/A | N/A |
| Ocular Stain positive (%) | 47 | N/A | N/A |
| Focus score (number of foci/4mm^2^ ), median (range) | 1.5 (0-12) | 3 (1-10) | N/A |
| Tarpley score, median (range) | 2 (0-4) | N/A | N/A |
| ANA≥1/320 (%) | 84.7 | N/A | N/A |
| Anti-Ro/SSA (%) | 75 | 87.1 | N/A |
| Anti-La/SSB (%) | 41 | 53.2 | N/A |
| RF positivity (>20 IU/ml) (%) | 56.6 | N/A | N/A |
| HGB (g/dL), median (range) | 12.8 (6.2-45.6) | 13.4 (11-15.6) | 13 (10.7-16.5) |
| ESR (mm/h), median (range) | 25 (3-140) | 20.5 (4-81) | N/A |
| Arthralgias-Myalgias (%) | 64.3 | N/A | N/A |
| Arthritis (%) | 17.9 | N/A | N/A |
| Raynaud's Phenomenon (%) | 26.2 | N/A | N/A |
| Palpable Purpura (%) | 17.3 | N/A | N/A |
| Lymphoma (%) | 23.3 | 8.6 | N/A |
| Hypergammaglobulinemia (>18%) (%) | 56.3 | 29.6 | N/A |
| TSH (mIU/L), median (range) | 1.3 (0.02-7.3) | 1.5 (0.02-3.9) | N/A |
| ESSDAI, median (range) | 9 (1-19) | N/A | N/A |
| ESSPRI, median (range) | N/A | 0.8 (0-1) | N/A |
| EDSS, median (range) | N/A | N/A | 1.5 (0-6) |

ANA: anti-nuclear antibodies, EDSS: Expanded Disability Status Scale, ESSDAI: EULAR Sjögren's syndrome disease activity index, ESSPRI: EULAR Sjogren's Syndrome Patient Reported Index, ESR: erythrocyte sedimentation rate, HGB: hemoglobin, N/A: not available, RF: rheumatoid factor, TSH: thyroid-stimulating hormone

**Supplementary Table 2.** Demographic, clinical, and laboratory characteristics according to fatigue status in the Greek primary SS cohort.

|  | **Fatigued**  **(n=64)** | **Non-fatigued**  **(n=135)** | **p-value** |
| --- | --- | --- | --- |
| **Demographic characteristics** |  |  |  |
| Female sex (%) | 98.5 | 92.6 | ns |
| Age at study entry (years), median (range) | 60 (20-85) | 59 (15-81) | ns |
| Age at primary SS diagnosis (years), median (range) | 51.5 (18-79) | 53 (15-81) | ns |
| **Clinical features** |  |  |  |
| **Local sicca symptoms** |  |  |  |
| Ocular dryness (subjective) (%) | 90.5 | 91.1 | ns |
| Oral dryness (subjective) (%) | 95.2 | 91.9 | ns |
| SGE (%) | 36.1 | 26.1 | ns |
| Abnormal Schirmer’s test (%) | 77.1 | 76.6 | ns |
| Ocular Stain positive (%) | 52.8 | 44.3 | ns |
| **Systemic symptoms** |  |  |  |
| Arthralgias-Myalgias (%) | 76.2 | 58.6 | 0.017 |
| Arthritis (%) | 19 | 17.3 | ns |
| Raynaud’s phenomenon (%) | 34.9 | 22.0 | 0.05 |
| Palpable purpura (%) | 15.9 | 18.0 | ns |
| ESSDAI, median (range) | 8.5 (1-16) | 9 (2-19) | ns |
| Lymphoma (%) | 30.2 | 20.0 | ns |
| **Laboratory features** |  |  |  |
| WBC number/mm^3^ (absolute number), median (range) | 5400 (3490-17700) | 5300 (2700-15400) | ns |
| Neutrophil number/mm^3^ (absolute number), median (range) | 3378 (1677-10443) | 3090 (865-13600) | ns |
| Lymphocyte number/mm^3^ (absolute number), median (range) | 1665 (350-5487) | 1500 (200-4380) | ns |
| Monocyte number/mm^3^ (absolute number), median (range) | 392 (35-1500) | 392 (94-1168) | ns |
| HGB (g/dL) (absolute number), median (range) | 12.6 (7.3-35.3) | 12.9 (6.2-45.6) | ns |
| ESR (mm/h), median (range) | 25 (5-100) | 24 (3-140) | ns |
| TSH (mIU/L), median (range) | 1.2 (0.4-5.0) | 1.4 (0.02-7.3) | ns |
| IgG (mg/dL), median (range) | 1455 (997-4760) | 1664 (783-5580) | ns |
| IgM (mg/dL), median (range) | 128 (23-389) | 113 (33-1370) | ns |
| IgA (mg/dL), median (range) | 258 (110-703) | 254 (90-800) | ns |
| LDH (U/L), median (range) | 226 (105-550) | 208 (113-511) | ns |
| C3 (mg/dl), median (range) | 105 (46-189) | 109 (32-165) | ns |
| C4 (mg/dl), median (range) | 18 (1-56) | 19 (2-48) | ns |
| Anti-Ro antibodies (%) | 73 | 75.9 | ns |
| Anti-La antibodies (%) | 33.3 | 44.7 | ns |
| *BAFF* mRNA levels (relative expression), median (range) | 1.8 (0.9-5.8) | 1.6 (0.2-5.6) | ns |
| *BAFF* mRNA levels (adjusted) (relative expression), median (range) | 1.3 (-0.6-13.2) | 1.3 (-1.1-7.2) | ns |
| Serum BAFF levels (pg/mL), median (range) | 1022 (462-2964) | 923 (425-6242) | ns |
| **Medications** |  |  |  |
| Steroids (%) | 45.3 | 26.7 | 0.009 |
| Hydroxychloroquine (%) | 57.8 | 41.5 | 0.03 |
| Anti-CD20 (%) | 28.1 | 14.1 | 0.02 |
| **Histopathological features** |  |  |  |
| Focus score (number of foci/4mm^2^), median (range) | 1.5 (0-12) | 1.5 (0-12) | ns |
| Tarpley score, median (range) | 2 (0-4) | 2 (0-4) | ns |

C3: complement C3, C4: complement C4, ESSDAI: EULAR Sjögren's syndrome disease activity index, ESR: erythrocyte sedimentation rate, HGB: hemoglobin, IgA: immunoglobulin A, IgG: immunoglobulin G, IgM: immunoglobulin M, LDH: lactate dehydrogenase, ns: non-significant, SGE: Salivary gland enlargement, TSH: thyroid-stimulating hormone, WBC: white blood cells

**Supplementary Table 3.** Associations of fatigue with psychological features in primary SS patients with or without fatigue (Greek cohort).

| **Phycological feature** | **Fatigued**  **(n=64)** | **Non-fatigued**  **(n=135)** | **p-value** |
| --- | --- | --- | --- |
| State anxiety >35 (%) | 95.3 | 60.9 | <0.001 |
| Trait anxiety >35 (%) | 96.8 | 70.4 | <0.001 |
| Depression >40 (%) | 84.4 | 51.9 | <0.001 |
| Athens Insomnia Scale >6 (%) | 90.3 | 41.4 | <0.001 |
| EPQP >2 (%) | 81.3 | 64.4 | 0.02 |
| EPQN >12 (%) | 92.2 | 49.6 | <0.001 |
| EPQE >9 (%) | 65.6 | 80 | 0.03 |

EPQE= Eysenck Personality Questionnaire extroversion; EPQN=EPQ neuroticism; EPQP= EPQ psychoticism; FACIT-F=Functional Assessment of Chronic Illness Therapy–Fatigue

**Supplementary Table 4.** Distribution of the allelic frequencies of five *BAFF* SNPs in the primary SS patients of the Greek cohort according to the presence or absence of fatigue.

| **SNP** | **Gene region** | **Location** | **Allele** | **Fatigued**  **(n=64)**  **n (%)** | **Non-fatigued**  **(n=135)**  **n (%)** | **HapMap/SNP database, %** | **OR [95%CI]** | **Fisher's**  **p value** |
| --- | --- | --- | --- | --- | --- | --- | --- | --- |
| **rs1224141** | intron | 107733724 | T  G | 100 (78.1)  28 (21.9) | 231 (85.6)  39 (14.4) | 85  15 | 1.659 [0.967-2.844] | 0.064 |
| **rs12583006** | intron | 107735453 | T  A | 91 (71.1)  37 (28.9) | 197 (73.0)  73 (27.0) | 76.1  23.9 | 1.097 [0.688-1.751] | 0.697 |
| **rs9514828** | promoter | 107719374 | C  T | 68 (53.1)  60 (46.9) | 120 (44.4)  150 (55.6) | 47  53 | 1.417 [0.929-2.161] * | 0.105 |
| **rs1041569** | promoter | 107717544 | A  T | 100 (78.1)  28 (21.9) | 203 (75.2)  67 (24.8) | 77  23 | 1.179 [0.714-1.947] | 0.521 |
| **rs9514827** | promoter | 107717404 | T  C | 88 (68.7)  40 (31.3) | 184 (68.1)  86 (31.9) | 69  31 | 0.973 [0.618-1.530] | 0.904 |

*Refers to the C allele

**Supplementary Table 5.** Demographic, clinical, and laboratory characteristics associated with the *BAFF* rs9514828 genotypes in the Greek primary SS cohort.

|  | **TT**  **(n=54)** | **CC/CT**  **(n=145)** | **p-value** |
| --- | --- | --- | --- |
| **Demographic characteristics** |  |  |  |
| Female sex (%) | 92.6 | 95.2 | ns |
| Age at study entry (years), median (range) | 59 (15-79) | 60 (20-85) | ns |
| Age at primary SS diagnosis (years), median (range) | 53 (15-76) | 52 (17-81) | ns |
| **Clinical features** |  |  |  |
| **Local sicca symptoms** |  |  |  |
| Ocular dryness (subjective) (%) | 96.3 | 88.9 | ns |
| Oral dryness (subjective) (%) | 92.6 | 93.1 | ns |
| SGE (%) | 28.3 | 29.6 | ns |
| Abnormal Schirmer’s test (%) | 81.4 | 75.0 | ns |
| Ocular Stain positive (%) | 38.5 | 49.4 | ns |
| **Systemic symptoms** |  |  |  |
| Arthralgias-Myalgias (%) | 68.5 | 62.7 | ns |
| Arthritis (%) | 14.8 | 19.0 | ns |
| Raynaud’s phenomenon (%) | 20.4 | 28.4 | ns |
| Palpable purpura (%) | 14.8 | 18.3 | ns |
| ESSDAI, median (range) | 9 (2-19) | 8.5 (1-17) | ns |
| Lymphoma (%) | 20.8 | 24.3 | ns |
| **Laboratory features** |  |  |  |
| WBC number/mm^3^ (absolute number), median (range) | 5500 (3020-17700) | 5300 (2700-15400) | ns |
| Neutrophil number/mm^3^ (absolute number), median (range) | 3393 (865-10443) | 3120 (1221-13600) | ns |
| Lymphocyte number/mm^3^ (absolute number), median (range) | 1500 (350-5487) | 1523 (200-4500) | ns |
| Monocyte number/mm^3^ (absolute number), median (range) | 394 (94-1500) | 390 (35-1168) | ns |
| HGB (g/dL) (absolute number), median (range) | 12.9 (6.2-15.1) | 12.8 (7.3-45.6) | ns |
| ESR (mm/h), median (range) | 30 (4-140) | 24 (3-130) | ns |
| TSH (mIU/L), median (range) | 1.3 (0.02-4.8) | 1.4 (0.02-7.3) | ns |
| IgG (mg/dL), median (range) | 1310 (783-2873) | 1640 (826-5580) | ns |
| IgM (mg/dL), median (range) | 135 (33-1370) | 117 (23-450) | ns |
| IgA (mg/dL), median (range) | 239 (90-399) | 261 (110-800) | ns |
| LDH (U/L), median (range) | 216 (113-502) | 213 (105-550) | ns |
| C3 (mg/dl), median (range) | 110 (36-154) | 107 (32-189) | ns |
| C4 (mg/dl), median (range) | 20 (5-47) | 18 (1-56) | ns |
| Anti-Ro antibodies (%) | 75.5 | 74.8 | ns |
| Anti-La antibodies (%) | 43.4 | 40.1 | ns |
| *BAFF* mRNA levels (relative expression), median (range) | 1.8 (0.7-5.8) | 1.8 (0.2-4.9) | ns |
| *BAFF* mRNA levels (adjusted) (relative expression), median (range) | 1.3 (-1.0-13.2) | 1.3 (-1.1-6.1) | ns |
| Serum BAFF levels (pg/mL), median (range) | 964 (558-4215) | 953 (425-6242) | ns |
| **Histopathological features** |  |  |  |
| Focus score (number of foci/4mm^2^), median (range) | 1.9 (0.4-12) | 1.5 (0-12) | ns |
| Tarpley score, median (range) | 2 (0-4) | 2 (0-4) | ns |

C3: complement C3, C4: complement C4, ESSDAI: EULAR Sjögren's syndrome disease activity index, ESR: erythrocyte sedimentation rate, HGB: hemoglobin, IgA: immunoglobulin A, IgG: immunoglobulin G, IgM: immunoglobulin M, LDH: lactate dehydrogenase, ns: non-significant, SGE: Salivary gland enlargement, TSH: thyroid-stimulating hormone, WBC: white blood cells
